# Diversity and Activity of Communities Inhabiting Plastic Debris in the North Pacific Gyre

**DOI:** 10.1128/mSystems.00024-16

**Published:** 2016-05-17

**Authors:** Jessica A. Bryant, Tara M. Clemente, Donn A. Viviani, Allison A. Fong, Kimberley A. Thomas, Paul Kemp, David M. Karl, Angelicque E. White, Edward F. DeLong

**Affiliations:** a Department of Civil and Environmental Engineering, Massachusetts Institute of Technology, Cambridge, Massachusetts, USA; b Daniel K. Inouye Center for Microbial Oceanography: Research and Education, University of Hawaii, Honolulu, Hawaii, USA; c Department of Oceanography, University of Hawaii, Honolulu, Hawaii, USA; d College of Earth, Ocean and Atmospheric Sciences, Oregon State University, Corvallis, Oregon, USA; Pacific Northwest National Laboratory

**Keywords:** North Pacific Gyre, biofilms, microbial communities, microplastics

## Abstract

Marine plastic debris has become a significant concern in ocean ecosystems worldwide. Little is known, however, about its influence on microbial community structure and function. In 2008, we surveyed microbial communities and metabolic activities in seawater and on plastic on an oceanographic expedition through the “great Pacific garbage patch.” The concentration of plastic particles in surface seawater within different size classes (2 to 5 mm and >5 mm) ranged from 0.35 to 3.7 particles m^−3^ across sampling stations. These densities and the particle size distribution were consistent with previous values reported in the North Pacific Ocean. Net community oxygen production (NCP = gross primary production − community respiration) on plastic debris was positive and so net autotrophic, whereas NCP in bulk seawater was close to zero. Scanning electron microscopy and metagenomic sequencing of plastic-attached communities revealed the dominance of a few metazoan taxa and a diverse assemblage of photoautotrophic and heterotrophic protists and bacteria. *Bryozoa*, *Cyanobacteria*, *Alphaproteobacteria*, and *Bacteroidetes* dominated all plastic particles, regardless of particle size. Bacteria inhabiting plastic were taxonomically distinct from the surrounding picoplankton and appeared well adapted to a surface-associated lifestyle. Genes with significantly higher abundances among plastic-attached bacteria included *che* genes, secretion system genes, and *nifH* genes, suggesting enrichment for chemotaxis, frequent cell-to-cell interactions, and nitrogen fixation. In aggregate, our findings suggest that plastic debris forms a habitat for complex microbial assemblages that have lifestyles, metabolic pathways, and biogeochemical activities that are distinct from those of free-living planktonic microbial communities.

**IMPORTANCE** Marine plastic debris is a growing concern that has captured the general public’s attention. While the negative impacts of plastic debris on oceanic macrobiota, including mammals and birds, are well documented, little is known about its influence on smaller marine residents, including microbes that have key roles in ocean biogeochemistry. Our work provides a new perspective on microbial communities inhabiting microplastics that includes its effect on microbial biogeochemical activities and a description of the cross-domain communities inhabiting plastic particles. This study is among the first molecular ecology, plastic debris biota surveys in the North Pacific Subtropical Gyre. It has identified fundamental differences in the functional potential and taxonomic composition of plastic-associated microbes versus planktonic microbes found in the surrounding open-ocean habitat.

**Author Video**: An author video summary of this article is available.

## Abstract (precis)

Marine plastic debris is a growing concern that has captured the general public’s attention. While the negative impacts of plastic debris on oceanic macrobiota, including mammals and birds, are well documented, little is known about its influence on smaller marine residents, including microbes that have key roles in ocean biogeochemistry. Our work provides a new perspective on microbial communities inhabiting microplastics that includes its effect on microbial biogeochemical activities and a description of the cross-domain communities inhabiting plastic particles. This study is among the first molecular ecology, plastic debris biota surveys in the North Pacific Subtropical Gyre. It has identified fundamental differences in the functional potential and taxonomic composition of plastic-associated microbes versus planktonic microbes found in the surrounding open-ocean habitat.

## INTRODUCTION

In the last decade, there has been a growing concern about the ecological impact of plastics in the marine environment. From 1950 to 2012, the rates of plastic production have increased by an average of 8.7% per year, with annual production rates nearing 300 million tons of plastic in 2013 (1, 2). A fraction of this material accumulates in the marine environment. Current estimates of the mass of plastic in the global ocean range from 7,000 to 300,000 tons (3, 4). This debris is found in all ocean basins, albeit not uniformly distributed. In 1988, scientists correctly predicted that buoyant plastic debris entering the ocean would become concentrated in regions termed “gyres,” where large-scale subtropical currents converge (5). This prediction has since been confirmed by multiple sampling efforts spanning the Pacific and Atlantic Ocean Gyres (3, 6–9).

While these gyres do not collect cohesive patches or floating islands of refuse, they are certainly zones where plastic debris is observed in elevated concentrations. The most well-publicized “patch,” the so called “great Pacific garbage patch,” is an accumulation zone roughly centered at 31°N, 139°W (10), where large-scale anticyclonic (clockwise) ocean circulation acts to trap and retain floating debris (6, 11). Despite the increasing research efforts to understanding the spatial distribution and temporal variance of marine plastic debris, the ecological implications of this refuse field are still largely unknown, particularly in regard to the potential consequences for lower tropic levels (e.g., phytoplankton and marine bacteria).

Plastic debris is known to impact marine organisms, including turtles, birds, mammals, fish, and invertebrates through entanglement and ingestion (12–14). There is also concern that some types of plastic debris are a source of toxic chemicals and/or adsorb persistent organic pollutants, including polychlorinated biphenyls, that could be biomagnified throughout the food chain (15–18). Additionally, a number of studies have clearly demonstrated that diverse biofouling organisms, such as bryozoans, settle on marine plastic debris (19–21). In this regard, plastic can serve as a vector for the introduction of nonnative species into new environments (22, 23). Small plastic particles, including those called microplastics (generally <5 mm in size, but see reference 24), may be particularly harmful, given that they are more abundant and that their reduced size makes them ingestible by small grazers that form the lower levels of the marine trophic system (24).

Despite known impacts of plastic on higher organisms, much less is known about the interactions between marine microbiota and plastic (25). Colonization of plastic particles by microbes was first reported in 1972 (26). Subsequent studies have shown that microbes rapidly colonize debris and that in the Atlantic Ocean, communities on plastic are taxonomically distinct from those in the surrounding water column (27–31). Little is known, however, about the nature of plastic-microorganism interactions, especially in the context of the entire biofouling community. More significantly, the potential for functional differences between microbes found on plastics and those in the surrounding water column has yet to be explored.

To address these uncertainties and to learn more about the nature of microbes that colonize plastics, we mounted the SUPER HI-CAT (Survey of Underwater Plastic and Ecosystem Response, Hawaii to California Transect) expedition to observe and sample plastic debris along a transect through the North Pacific Subtropical Gyre (NPSG) in 2008. We hypothesized that microplastics in the Pacific plastic patch harbor communities that (i) are metabolically active, with productivity and respiration rates that differ in magnitude from equivalent volumes in the surrounding water column; (ii) are taxonomically distinct from free-living picoplankton but similar to plastic-attached communities sampled in the Atlantic Ocean; and (iii) have protein-coding genes that differ from those of the surrounding free-living picoplankton.

## RESULTS

### Concentration and size distribution of plastic fragments.

**FIG 1:**
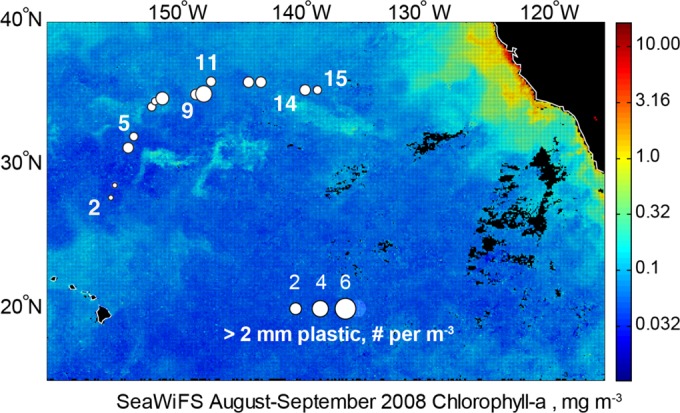
Locations of sampling stations along our transect. The area of each circle corresponds to the concentration of plastic particles with a diameter of >2 mm. Station numbers are next to the stations where samples used for molecular analyses were collected. Composite satellite SeaWiFS measurements of sea surface Chl *a* (up to a depth of approximately 25 m) from August and September 2008 are shown for context. For reference, the center of the NPSG accumulation zone is at 31°N, 139°W (10).

**FIG 2:**
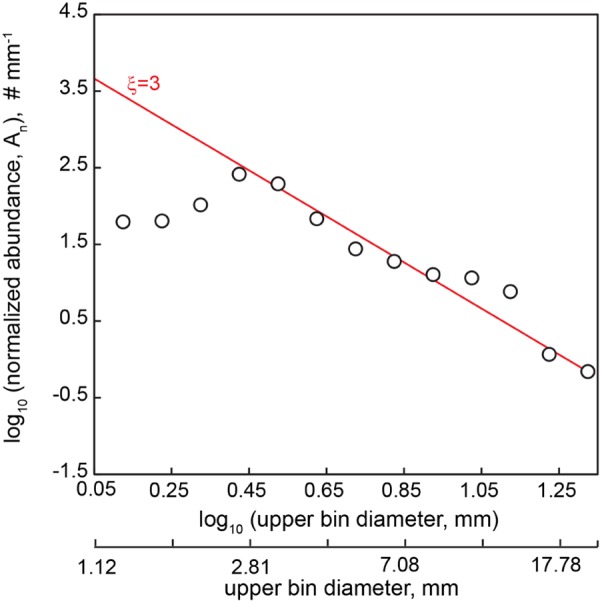
Size distribution of 554 microplastic particles collected in the NPSG in August 2008. Bins are spaced 0.1 log unit apart, and the *x* axis represents the upper edge of these logarithmic bins. For reference, the bin diameter in millimeters is also shown. The relationship between particle diameter and particle abundance normalized to bin width (A_n_) is characterized by a power law with an exponent (*ξ*) equal to 3 (red line). The particle size distribution of microplastic collected in the NPSG adheres to this fit at diameters of >3 mm.

Plastic fragments were recovered from 14 manta trawls carried out between the Hawaiian Islands and California (Fig. 1; see Table S1 in the supplemental material). A subset of these particles (three particles in each of the two larger size classes and four particles in the 0.2- to 2-mm size class) was analyzed by Fourier transform infrared spectroscopy (FTIR) and confirmed to be composed of either polyethylene or polypropylene polymers. The concentration of plastic encountered along this transect varied by an order of magnitude. For the two largest size classes sampled by the manta trawl (>2 mm), surface concentrations ranged from 0.35 to 3.7 fragments/m^3^, with the highest values (3.71 pieces/m^3^) recorded at approximately 35°N, which is roughly the position of the subtropical front (Fig. 1). When integrated over the upper 0.15 m of the water column, neustonic plastic concentrations ranged from 51,000 to 556,500 fragments/km^2^ of sea surface (sum of >2- to 5-mm and >5-mm particles). For reference, analysis of existing plastic concentrations in other studies in the NPSG (data from 1972 to 2012) ranged from 18,160 to 557,700 pieces/km^2^ (see the summaries of Law et al. [9] and Goldstein et al. [32]). The distribution of plastic particle sizes was reasonably modeled with a power law scaling exponent in the size bins above 3 mm, but below which plastic concentrations begin to decrease (Fig. 2).

10.1128/mSystems.00024-16.9 Table S1  Sampling station information and summary of plastic concentrations in the >2- to 5-mm and >5-mm size classes at each station in the NPSG. Download Table S1, DOCX file, 0.1 MB. Copyright © 2016 Bryant et al. 2016 Bryant et al. This content is distributed under the terms of the Creative Commons Attribution 4.0 International license.

### Biotic activity on microplastics.

**FIG 3:**
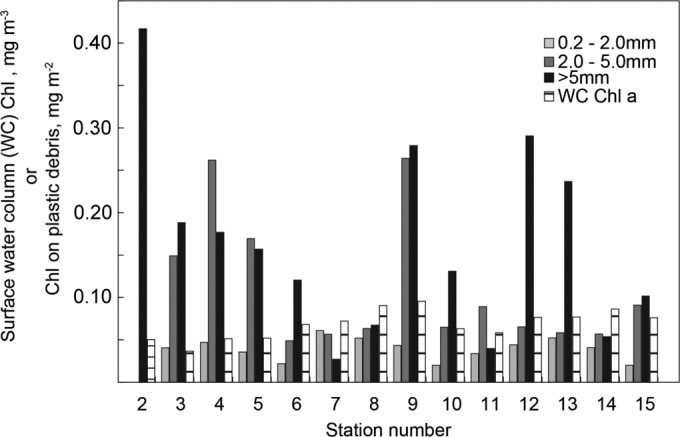
Chl *a* concentrations on the three size classes of plastic debris and in the surrounding surface water at each station.

**FIG 4:**
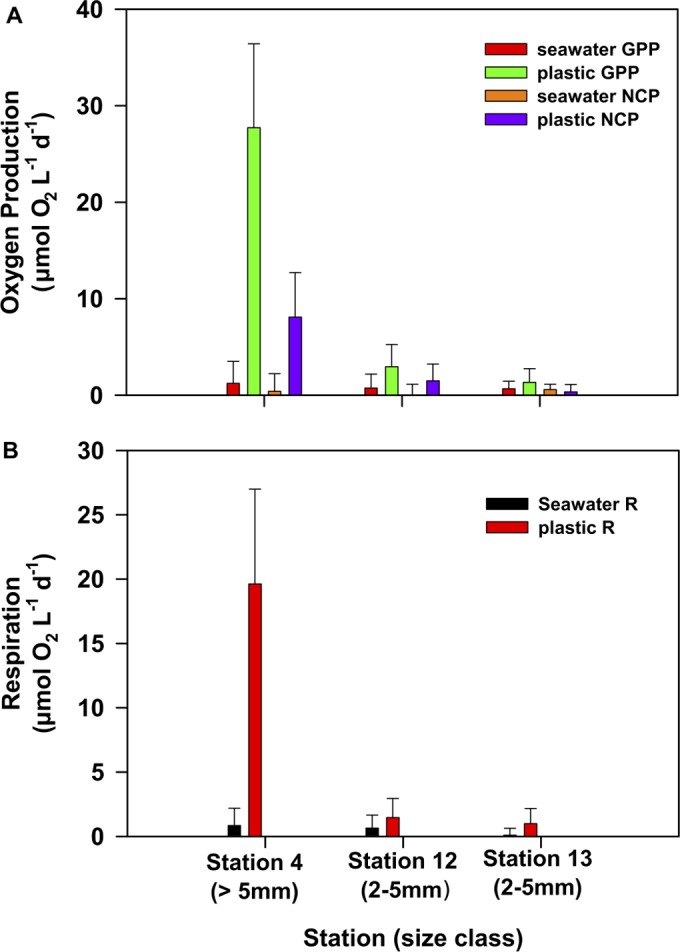
GPP, NCP (A), and R (B) rates of the communities attached to plastic particles and in the surrounding surface seawater, measured by using oxygen fluxes. GPP was calculated as the sum of NCP and R. Bars represent standard deviations.

Chlorophyll *a* (Chl *a*) measurements, combined with oxygen production and respiration measurements, demonstrated that metabolically active photosynthetic and heterotrophic organisms were attached to plastic debris (Fig. 3 and 4). Chl *a* concentrations measured on the plastic debris ranged from approximately 0.03 to 0.42 mg/m^2^, while Chl *a* concentrations in the surrounding seawater ranged from approximately 0.04 to 0.1 mg/m^3^. Assuming the water column Chl *a* concentrations we measured at each station and the Chl *a* concentrations on the >5-mm plastic particles, a spherical plastic particle with a diameter of 5 mm contains the same amount of Chl as approximately 30 to 700 ml of seawater.

The concentrations of Chl *a* scaled to surface area were also higher on larger pieces of plastic (Fig. 3). Chl concentrations on the three size classes differed significantly (Kruskal-Wallis rank sum test; *P* < 0.05), and a *post hoc* Dunn test showed that the >5-mm and 2- to 5-mm size classes had significantly higher Chl concentrations than the 0.2 to 2-mm size class (false-positive rate [FDR], <0.05).

We estimated bulk community metabolic rates on plastic and in the surrounding seawater in terms of net oxygen production (net community production [NCP]), total oxygen consumption (community respiration [R]), and total oxygen production (gross primary production [NCP + R = GPP]). The >5-mm particle size fraction NCP and R rates were significantly higher than the seawater rates (Mann-Whitney U test, *P* < 0.01). In addition, both experiments using >2- to 5-mm size fraction pieces had greater R than seawater (one-way analysis of variance [ANOVA], *P* < 0.05), and at station 12, the >2- to 5-mm size fraction pieces demonstrated greater rates of NCP than seawater (one-way ANOVA; *P* < 0.05). Seawater amendments with the smallest size fraction particles resulted in production and respiration values that were similar to those of unamended water samples (data not shown).

### Eukaryotic and prokaryotic organisms on microplastics.

**FIG 5:**
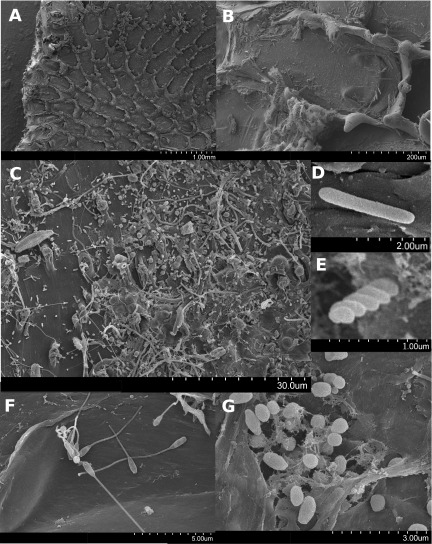
SEM images of organisms on microplastic particle surfaces. A scale bar is located at the bottom right of each image with the value designating the length of the entire scale bar. (A) A bryozoan colony on the surface of a plastic particle. (B) An individual bryozoan zooid with diatom-shaped organisms attached to its operculum. (C) Region of a bryzoan zooid frontal membrane densely covered with cells of various phenotypes. (D to G) Cells on the surfaces of plastic particles.

Inspection of >5-mm plastic particles collected from stations 2, 14, and 15 by scanning electron microscopy (SEM) revealed that samples were heavily colonized by encrusting bryozoans (Fig. 5). In particular, the frontal membranes of the bryozoans were associated with multispecies microbial biofilms that included pennate diatoms, as well as coccus-, rod-, and spiral-shaped cells. Bacterial cells with prosthecae and long filaments were also observed on bryozoan surfaces. Similar cell morphologies were seen directly on the surfaces of the plastic particles, with some cells nested within pores in the plastic.

We extracted DNA from communities attached to 12 plastic particles collected across the oceanographic transect and analyzed the DNA by metagenomic shotgun sequencing (here, referred to as metagenomic samples). Metagenomic sample numbers correspond to sampling station numbers as in Fig. 1. The letters a and b indicate particles from the >5-mm and 2- to 5-mm sample size classes, respectively. We used the paired-end reads within our metagenomic libraries that mapped to small-subunit (SSU) rRNA genes in the SILVA database to identify the taxonomic origins of organisms in our samples. Between 40 and 99% of the reads in each sample that mapped to SSU rRNA genes mapped to eukaryotic SSU rRNAs, with the remainder mapping to bacteria (see Fig. S1 in the supplemental material). Some, but not all, eukaryotic and bacterial communities on plastic particles from the same station clustered together in nonmetric multidimensional scaling (NMDS) plots (see Fig. S2 in the supplemental material).

10.1128/mSystems.00024-16.2 Figure S1  Bar chart displaying the relative abundances of *Eukaryota*, *Archaea*, and *Bacteria* within reads from plastic particle metagenomic libraries mapping to SSU rRNA genes. Reads were assigned to the LCA of top hits to the SILVA database. Download Figure S1, TIF file, 2.6 MB. Copyright © 2016 Bryant et al. 2016 Bryant et al. This content is distributed under the terms of the Creative Commons Attribution 4.0 International license.

10.1128/mSystems.00024-16.3 Figure S2  Nonmetric multidimensional scaling plots visualizing Bray-Curtis distances between eukaryotic (A) and bacterial (B) communities. Symbol shapes and colors indicate the stations from which the samples originated. The size of each symbol indicates the size fraction, with the larger symbol indicating the >5-mm class and the smaller symbol indicating the >2- to 5-mm size class. The image was generated by using reads mapping to SSU rRNA genes that could be assigned to the class (A) or family (B) taxonomic level by the LCA approach. Download Figure S2, EPS file, 0.1 MB. Copyright © 2016 Bryant et al. 2016 Bryant et al. This content is distributed under the terms of the Creative Commons Attribution 4.0 International license.

**FIG 6:**
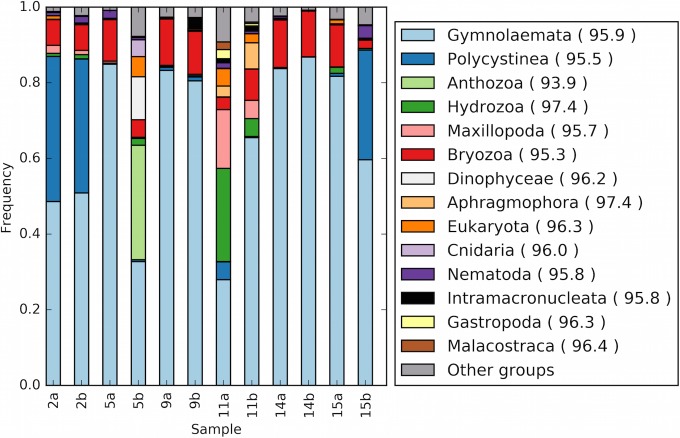
Bar chart displaying the abundance of eukaryotic classes within reads mapping to SSU rRNA genes. Reads were assigned to the LCA of top hits to the SILVA database. Clade abundances in each sample are relative to the total number of reads per sample mapping to a eukaryotic SSU rRNA gene. Clades with abundances of >1% in at least one sample are shown. The average percent identities of sample reads to their top hit within each taxonomic group are displayed in parentheses.

Consistent with the SEM images, between 30 and 90% of the eukaryotic SSU rRNA gene reads from all 12 plastic particles mapped to bryozoan rRNA genes (Fig. 6). In addition, samples 2a, 2b, and 15b also harbored a high abundance of polycystine radiolarians and a large percentage of reads from 11a and 11b mapped to *Hydrozoa*, *Maxillopoda*, and *Aphragmophora* database sequences. Sample 5b also contained a high abundance of both *Dinophyceae* and *Anthozoa*.

Diatom clades did not make up more than 1% of the eukaryotic SSU rRNA genes in any of our metagenomic libraries, despite being evident in SEM images (see Fig. S3 in the supplemental material) and being frequently abundant on plastic debris in other studies (21, 29, 33–35). Their low representation in the metagenomic samples may be due to their low biomass (as opposed to the number of individuals) compared to the other eukaryotes present. Between 10 and 50% of the reads mapping to chloroplast rRNA genes did map to diatom clades (see Fig. S4 in the supplemental material). Other chloroplast sequences mapped to algal classes, including *Stylonematophyceae*, *Filosa-Chlorarachnea*, and *Pelagophyceae* (see Fig. S4 in the supplemental material).

10.1128/mSystems.00024-16.4 Figure S3  Additional diatom SEM images. (A) Diatoms nested within a crevice on the surface of a plastic particle. (B) Diatoms attached to a bryozoan frontal membrane. (C) Diatoms attached to bryozoan tentacles. Download Figure S3, TIF file, 18.8 MB. Copyright © 2016 Bryant et al. 2016 Bryant et al. This content is distributed under the terms of the Creative Commons Attribution 4.0 International license.

10.1128/mSystems.00024-16.5 Figure S4  Bar chart displaying the relative abundance of photosynthetic eukaryotic classes within reads mapping to chloroplast SSU rRNA genes. The PhytoREF database was used for taxonomic assignments, and abundances are relative to the total number of reads in each sample mapping to sequences in the PhytoREF database. The average percent identities of sample reads to their top hit within each taxonomic group are in parentheses to the right of clade names. The percentages of total SSU rRNA gene reads mapping to chloroplasts in the SILVA database are in parentheses next to the sample names on the *x* axis. Download Figure S4, TIF file, 2.3 MB. Copyright © 2016 Bryant et al. 2016 Bryant et al. This content is distributed under the terms of the Creative Commons Attribution 4.0 International license.

10.1128/mSystems.00024-16.6 Figure S5  Bar chart displaying the relative abundances of bacterial classes within reads mapping to SSU rRNA genes. Reads were assigned to the LCA of their top hits to the SILVA database. Clade abundances in each sample are relative to the total number of reads per sample mapping to bacterial SSU rRNA genes. Taxonomic groups with abundances of >3% in at least one sample are shown. The average percent identities of sample reads to their top hits within each taxonomic group are in parentheses. Download Figure S5, TIF file, 2 MB. Copyright © 2016 Bryant et al. 2016 Bryant et al. This content is distributed under the terms of the Creative Commons Attribution 4.0 International license.

**FIG 7:**
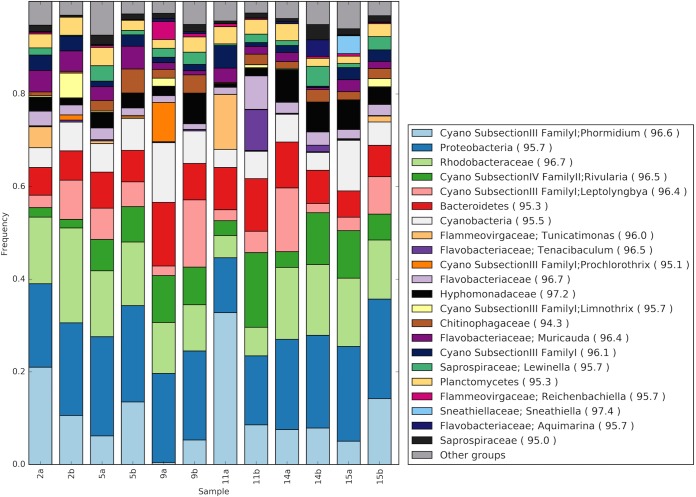
Bar chart showing the relative abundance of prokaryotic groups based on reads mapping to prokaryotic SSU rRNA genes. Reads were assigned to the LCA of top hits to the SILVA database. Where possible, reads were assigned prokaryotic genera. Broader taxonomic groups are made up of reads that could not be assigned to a genus or had abundances of <3% in all libraries. Clade counts in each sample were normalized to the total number of SSU rRNA gene reads mapped to bacterial taxa in each sample. The average percent identities of sample reads to their top hit within each taxonomic group are in parentheses.

Bacterial SSU rRNA genes revealed that *Cyanobacteria* and *Alphaproteobacteria* were consistently among the most abundant prokaryotic groups on plastic particles. *Flavobacteriia*, *Cytophagia*, *Sphingobacteriia*, *Gammaproteobacteria*, and *Deltaproteobacteria* were also present across all of the samples (see Fig. S4 in the supplemental material). Consistent with the filaments observed in the SEM images, higher-resolution taxonomic assignments showed that the most abundant *Cyanobacteria* across the 12 samples were most closely related to the filamentous genera *Phormidium*, *Rivularia*, and *Leptolyngbya. Rhodobacteraceae*, and *Hyphomonadaceae*, the latter having prosthecae, appendages likes those in Fig. 5F, were among the most abundant alphaproteobacterial groups (Fig. 7). The *Bacteroidetes* genera “*Tunicatimonas*” and *Tenacibaculum* each made up approximately 10% of one sample, while *Muricauda* and *Lewinella* were identified at lower abundances across the samples.

**FIG 8:**
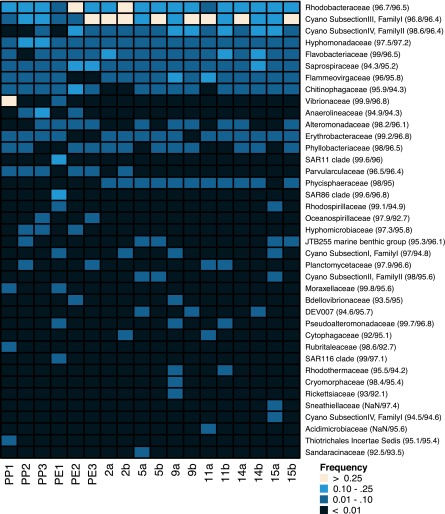
Heat map displaying the abundances of bacterial families identified on plastics particles collected from the North Atlantic Subtropical Gyre (NASG, samples PP1 to PE3; abbreviations: PP, polypropylene; PE, polyethylene) (29) and the NPSG (samples 2a to 15b). The abundance of each bacterial family in a sample is relative to the total number of SSU rRNA gene reads in that sample assigned a prokaryotic family. Families with abundances of >1% in at least one sample are shown. The average percent identities of sample reads to their top hit (NASG/NPSG) are in parentheses. Cyano, *Cyanobacteria*.

Zettler et al., who used amplicon sequencing to characterize the bacterial communities on three pieces of polyethylene and three pieces of polypropylene collected in the oligotrophic North Atlantic Subtropical Gyre in a recent study, reported observing similar bacterial taxa (29). We reannotated their amplicon data by using our work flow for our (unamplified) metagenomic rRNA sequences in order to directly compare our SSU rRNA gene data with theirs. The *Cyanobacteria* subsection III family I group, which includes *Phormidium* and *Leptolyngbya*, and *Rhodobacteraceae* were the most abundant microbial families within and across both studies (Fig. 8). *Hyphomonadaceae*, *Flavobacteriaceae*, *Saprospiraceae*, and *Flammeovirgaceae* were also consistent members of the microbial plastic communities. Overall, the most abundant groups in the previous study and those reported here were strikingly similar. The one major exception was *Vibrionaceae*, which had a very high abundance in one sample from the Atlantic but was otherwise not common in samples from either study.

### Taxonomic and functional gene comparison to surrounding seawater.

The bacterial taxa making up the plastic-associated microbial communities were distinct from the previously well-characterized free-living clades found in the surrounding seawater in the NPSG, which are consistently dominated by the oligotrophic *Prochlorococcus* (*Cyanobacteria* subsection I, family I) and SAR11 clades (36–38). To illustrate this, we compared the microbial families found in our plastic samples to 17 metagenomic libraries from free-living picoplankton surface water microbial communities collected in the NPSG over a 2-year period bracketing the dates when our plastic samples were collected. Only two clades, *Rhodobacteraceae* and *Flavobacteriaceae*, made up at least 1% of the reads mapping to SSU rRNA genes in more than half of the samples from either community. However, both were significantly more abundant in the plastic-associated communities (see Fig. S6 in the supplemental material; FDR, <0.005). In addition, bacterial family richness was higher in plastic samples than in planktonic samples (*P* < 0.001; see Fig. S7 in the supplemental material).

10.1128/mSystems.00024-16.7 Figure S6  Heat map comparing abundances of prokaryotic families identified in the NPSG planktonic samples (H197 to H215) and plastic-associated samples (2a to 15b). The abundance values are relative to the total number of bacterial SSU rRNA reads in the sample assigned a prokaryotic family and have been rounded to the hundredth decimal place (unlike in Fig. 8). Families with at least 1% abundance in one sample are shown. The average percent identities of sample reads to their top hit within each family (planktonic/plastic associated) are in parentheses. An asterisk next to a clade name indicates significantly different relative abundances in the two habitats (FDR, <0.005). Abbreviations: Cyano, *Cyanobacteria*; Gamma, *Gammaproteobacteria*. Download Figure S6, TIF file, 18.1 MB. Copyright © 2016 Bryant et al. 2016 Bryant et al. This content is distributed under the terms of the Creative Commons Attribution 4.0 International license.

10.1128/mSystems.00024-16.8 Figure S7  Bar chart displaying the average numbers of bacterial families identified in NPSG planktonic and plastic-associated samples. Family abundances in each sample were calculated as described in the legend to Fig. S6, and families were required to have an abundance of >1% to be counted as present in a sample. Plastic-associated samples had significantly more families than NPSG samples (Welch’s two-sample *t* test, *P* < 0.001). Bars outline 95% confidence intervals. Download Figure S7, TIF file, 2.6 MB. Copyright © 2016 Bryant et al. 2016 Bryant et al. This content is distributed under the terms of the Creative Commons Attribution 4.0 International license.

**FIG 9:**
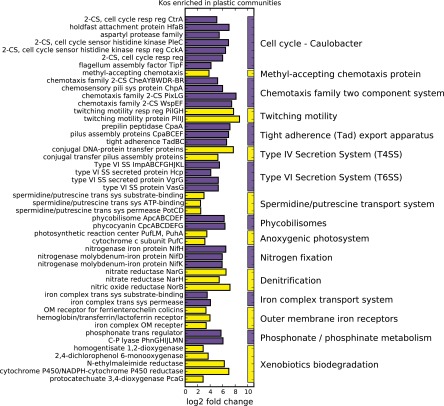
Selected KEGG genes that were significantly more abundant (>2 log_2_-fold change; FDR-adjusted *P* < 0.005) in plastic-associated metagenomic libraries than in the picoplankton community in the surrounding water column. Some gene-encoded products with related functions have been condensed, and the log_2_-fold change has been averaged (e.g., *phnGHIJLMN*). For a list of all of the KEGG genes identified in this study and a list of the KEGG genes and descriptions included here, see Data Set S1 in the supplemental material. Abbreviations of gene product descriptions: 2-CS, two-component system; resp reg, response regulator; OM, outer membrane; SS, secretion system; sys, system; trans, transport.

To better understand how these taxonomic differences correspond to differences in the functional gene repertoires, we compared the abundances of KEGG orthologs (KOs) found in our plastic-attached communities with those in free-living communities. Of the 5,912 KOs tested, 18% (1,064) were at least four times as abundant in the plastic-attached metagenomes, while only 2% (129) were more abundant in the water column (FDR, <0.005; see Data Set S1 in the supplemental material). Of the KOs with significantly different abundances, as determined by the DESeq2 algorithm, only 13 were not detected as significantly different by Mann-Whitney U tests (FDR, <0.005; see Data Set S1 in the supplemental material). The difference in KO abundances between communities, in part, simply reflects the larger genomes associated with abundant taxa on the plastic fragments. It also indicates the enrichment of taxa with specific metabolic pathways and genes (secretion systems, nitrogen fixation, motility, etc.) in plastic-attached versus free-living communities (Fig. 9; see the Discussion).

10.1128/mSystems.00024-16.1 Data Set S1  Full results of the DESeq2 comparison of plastic-associated and picoplankton bacterial KEGG genes. The log_2_-fold change of each KO was tested only once but may be listed multiple times, depending on the number of KEGG categories it is assigned to in the KEGG BRITE functional hierarchy (columns B to F). Columns G to K display the results of the DESeq2 analysis. Columns L to AN display the number of reads in each sample mapping to each KO. The symbol ^ in column D indicates that a KO determined to be significantly differentially abundant by the DESeq2 algorithm was not also found to be significantly differentially abundant by a Mann-Whitney U test (FDR, <0.005).The list of selected KEGG genes used in Fig. 9 is also included. Download Data Set S1, XLSX file, 4.7 MB. Copyright © 2016 Bryant et al. 2016 Bryant et al. This content is distributed under the terms of the Creative Commons Attribution 4.0 International license.

## DISCUSSION

Plastic particle densities across our transect were of the same order of magnitude (52,233 to 556,152 particles/km^2^) as those previously reported in the North Pacific (4, 9, 32). In addition, our particle size distribution was strikingly consistent with previous observations. Cózar et al. (3) predicted that the fractal nature of plastic fragmentation should result in the smallest plastic size classes having the largest number of particles and that a steady-state plastic abundance-size distribution should follow a power law with a scaling exponent of 3. However, consistent with other studies (4), Cózar et al. only observed a power law relationship for size classes greater than approximately 5 mm, followed by a steep decline in plastic concentrations in the smaller size classes. We observed the same decline in the smallest size classes. Although we were careful to separate plastic particles from organic matter during sampling, we cannot rule out the possibility that we underestimated small plastic particles with diameters between 1 and 3 mm. A recent study also suggested that smaller particles may be distributed across deeper ocean depths than larger particles and therefore be captured at lower rates by surface trawls (39). Alternatively, as suggested in previous studies, small plastic particles may be selectively lost from the upper ocean due to unknown processes (3, 4).

The high density of Chl *a* we observed on plastics, combined with the high oxygen production measurements relative to the surrounding water column, suggests that larger microplastic particles are creating net autotrophic “hot spots” in the oligotrophic ocean. Determination of the exact source of the increased oxygen production and respiration rates is complicated by potentially enhanced activity of planktonic organisms surrounding the plastic particles during incubation. The biofilms on microplastics are the most likely source, however, given the short incubation times, as well as the high density of Chl *a* and the diverse array of eukaryotic and prokaryotic organisms observed on microplastics.

Our findings are consistent with earlier work demonstrating that plastics, in particular microplastics, harbor a distinct biota and represent a new habitat for rafting organisms in the NPSG, especially within accumulation zones (3, 4, 20). The eukaryotic groups we observed in our metagenomic libraries (including *Anthozoa*, *Hydrozoa*, *Maxillopoda*, and *Aphragmophora*) have all been reported in association with rafting communities on either natural (e.g., macroalgae, wood, pumice) or artificial substrates (20, 22, 34, 40). Encrusting bryozoans, in particular, have been reported as abundant organisms in previous marine debris surveys, including in the NPSG (19–22, 40).

It was intriguing that radiolarians were observed in such high abundances in three of our metagenomic samples, since they have been visually observed in low abundances in only one previous debris study (35) but were also observed on plastic debris by molecular approaches by Zettler et al. (29). This may represent radiolarian “by-catch” in the plankton net tows, as opposed to true association with microplastic particles. Also of interest was the co-occurrence of both *Dinophyceae*, most similar to *Symbiodinium* spp., and *Anthozoa* in sample 5b, suggesting that coral and the photosynthetic dinoflagellate symbionts may sometimes occupy this niche.

Consistent with previous marine plastic debris work in different systems, the bacterial taxa we observed on plastic particles are strikingly different from the clades known to reside in the surrounding water column (29, 30). In the NPSG, *Prochlorococcus* spp. are the most abundant planktonic cyanobacteria, consistent with Fig. S6 in the supplemental material (41). In addition, previous studies in tropical and subtropical waters have shown that *Trichodesmium* and *Crocosphaera* species represent the major diazotrophic cyanobacteria (42, 43). We did not observe these two taxa in this study, at least in part because they would have been excluded by the sampling prefilters used to generate the planktonic data. Regardless, these and other known open-ocean marine cyanobacteria (43) differ from the abundant cyanobacteria (*Phormidium*, *Leptolyngbya*, *Prochlorothrix*, and *Rivularia*) we observed in the plastic microbiota. *Phormidium* and *Rivularia* have also been observed on marine plastic debris in subtropical Atlantic and Northern European waters, as well as mats and benthic environments (29, 30, 44). *Prochlorothrix* cyanobacteria, to our knowledge, have previously been identified only in fresh or brackish water (45). Similarly, the bacterial clades “*Tunicatimonas*,” *Tenacibaculum*, *Hyphomonadaceae*, *Chitinophagaceae*, *Muricauda*, and *Lewinella* are not commonly observed as planktonic heterotrophs in the NPSG (36–38). At the family level, *Rhodobacteraceae* and *Flavobacteriaceae* were the only abundant clades found on our plastic metagenomic samples that are also reported in open-ocean picoplankton communities, including the NPSG (see Fig. S6 in the supplemental material) (38, 46, 47).

The clades we observed associated with microplastics all appear well adapted to take advantage of niches created by surfaces. The abundant bacterial families we found on our microplastic samples, which were separated by as much as 1,700 km in the NPSG, were consistent with the clades observed on plastic debris in the Atlantic Ocean. These observations suggest that a predictable core group of clades occupies the niche created by small plastic debris in oligotrophic surface waters worldwide.

With respect to particular taxonomic groups, members of the *Rhodobacteraceae* clade are known to alternate between diverse lifestyles (e.g., planktonic and attached) and are also capable of rapid responses to various resources (48, 49). It is likely that these characteristics explain their observed frequency as early colonizers of artificial surfaces, including glass, polyvinyl chloride, and Plexiglas surfaces, and explain why we observed them in high abundances across our samples (27, 50, 51). Likewise, the marine *Bacteroidetes* groups *Flavobacteriaceae* and *Saprospiraceae* have a known preference for growth on particles, surfaces, and algae (52, 53) and members of the family *Hyphomonadaceae* are considered oligotrophs that readily form biofilms on surfaces (54). Together, these findings suggest that the microbial communities we observed seem more indicative of a general proclivity for surface attachment, as opposed to any specific selection of microbiota by the chemical composition of the plastic substratum itself.

We were unable to determine which bacteria might have been attached to the eukaryotic organisms (like the *Bryozoa* that can cover much of the substrate surface area) versus directly attached to the plastic substrate. Many of the bacterial groups we observed have been previously documented living in association with eukaryotic organisms, including marine invertebrates, corals, and sponges (55–57). The genus “*Tunicatimonas*,” which we observed was most abundant on the plastic particle with the highest number of hydrozoan reads, was first isolated from a sea anemone (58). The *Flavobacteria* clade *Tenacibaculum* (also observed by Zettler et al. [29] and Oberbeckmann et al. [30]) has been isolated from a variety of marine organisms, including bryozoans (59), and a few members of this genus are known fish pathogens (60, 61). *Leptolyngbya* and *Phormidium* sp. strains have been identified as members of the coral black band disease consortium (62).

The large number of KOs that were significantly more abundant in plastic-associated metagenomes than in free-living metagenomes provided further evidence that microplastics create a niche that is distinct from the niches utilized by the surrounding picoplankton. While it is possible that some differences are a result of differences in the sequencing technologies used to generate data from these two communities, previous studies suggest that this is unlikely. For example, a previous study comparing the 454 and Illumina platforms in an aquatic system showed that both platforms sample the same fraction of diversity and produce similar relative abundances of genes and genomes (63). Future gene or protein expression studies will provide additional information on the potential importance of the functions of these genes in plastic-associated habitats.

Not surprisingly, our results suggest that the microbial communities that develop on the plastics are enriched for traits necessary for a surface-attached lifestyle. Consistent with the stalked cells observed in the SEM image and the presence of *Hyphomonadaceae* across our samples, many of the *Caulobacter*-like cell cycle genes that are involved in transitions between a flagellated motile lifestyle and a sessile cell with a prosthecum were significantly more abundant in the plastic metagenomes (54) (Fig. 9; see Data Set S1 in the supplemental material). Similarly, methyl-accepting chemotaxis protein-encoding genes, the majority of the two-component system CheA family (chemotaxis-like) genes, the *potABCD* spermidine/putrescine transporter components, and KOs within the tight adherence export apparatus system were all more abundant in the plastic-associated metagenomes. These genes have been implicated in chemotaxis, signaling of swarming activity, surface motility, colonization, and biofilm formation (64–68) (Fig. 9; see Data Set S1 in the supplemental material).

KOs belonging to secretion system pathways, including numerous type IV secretion system (T4SS) genes and the majority of T6SS components were also more abundant in the plastic metagenomes. The most common function of T4SSs is to conjugate plasmid DNA, and hence, T4SS plays an important role in gene flow between cells (69). The T6SS transports effector proteins directly into neighboring eukaryotic or prokaryotic cells, thereby playing a key role in competition or pathogenesis (70). T4SSs have also been shown to mediate the transfer of toxins and other effector proteins in several pathogens.

Concerns have been raised that plastic debris could transport pathogens or other unfavorable organisms, including dinoflagellates that cause harmful algal blooms (23, 29). The presence of bacterial clades with some pathogenic members has been interpreted by some as evidence that plastic debris may act as a disease vector (29). We cannot, however, draw any definitive conclusions in this regard. Potentially pathogenic species, for example, frequently contain strains that are benign. Additionally, secretion systems are also used in many other processes, including nonpathogenic, nontoxic interbacterial interactions. Finally, little is known about the natural distribution and dispersal mechanisms of many pathogenic and nonpathogenic marine microbes and traits, so it is difficult to postulate how plastic debris impacts these natural processes (70, 71).

The significantly higher abundance of phycobilisome antenna protein-encoding genes in the plastic metagenomes compared to the increase of some Chl *a*/*b*-binding light-harvesting protein-encoding genes in the surrounding water column shows that the dominant cyanobacteria in the two habitats use different light-harvesting machinery (Fig. 9; see Data Set S1 in the supplemental material). The majority of cyanobacteria are believed to absorb photons for photosynthesis by using phycobilisome complexes, while *Prochlorococcus* bacteria, the dominant cyanobacteria in the surrounding water column, utilize Chl-binding complexes. It has been postulated that *Prochlorococcus* bacteria evolved the alternative light-harvesting mechanism to cope with the limited nutrients, including iron and nitrogen, in oligotrophic gyres (72). Assuming this, the prevalence of phycobilisome-utilizing cyanobacteria on plastics, in combination with the elevated rates of oxygen production and respiration on plastics relative to that in the background seawater, suggests that the nutrient limitation in the NPSG is less severe in plastic particle communities.

Consistent with this, the increased abundance of nitrogenase genes *nifH*, *nifD*, and *nifK* in the plastic-associated metagenomes suggests that nitrogen fixation could be reducing nitrogen limitation on the plastics. Additionally, key enzymes involved in phosphonate utilization were more abundant in plastic communities. Phosphonates are increasingly being recognized as an important source of phosphorus (73–76), and it has been suggested that some microbes use phosphonates when nitrogen fixation relieves nitrogen limitations (76). This is consistent with the possibility that oligotrophic conditions are reduced on plastic particles, at least in relation to nitrogen. Future studies focusing on biomass accumulation and nutrient fluxes on microplastics will clarify the extent to which microplastics are creating a eutrophic niche in oligotrophic waters.

A large number of membrane transporters were significantly more abundant on plastics. Notably, three genes encoding TonB-dependent iron complex outer membrane receptors that import chelated iron and two genes forming an inner membrane iron complex transport system involved in siderophore import were more abundant in plastic metagenomes (77, 78). Siderophore uptake is important to community dynamics on large marine particles, and siderophore biosynthesis is important for biofilm maturation in some taxa (79, 80).

Whether or not the microorganisms residing on plastic debris are degrading plastics and significantly contributing to the loss of plastic from marine surface waters is an ongoing question (81). It has been hypothesized that microbial communities associated with plastic debris could also be degrading organic pollutants adsorbed to plastic debris, as biofilms are often involved in remediation processes (81, 82). Similar to Zettler et al. (29), we observed SSU rRNA genes related to bacterial clades with hydrocarbon-degrading members or members that have sometimes been associated with oil-contaminated environments, including *Phormidium*, *Muricauda*, *Hyphomonadaceae*, and *Rhodobacteraceae* (54, 83–85). It has also been suggested that some *Rhodobacteraceae* strains isolated from coastal environments are capable of lignin degradation, an activity that may be associated with plastic degradation (86, 87).

Several putative xenobiotic biodegradation genes were more abundant on plastic particles, including homogentisate 1,2-dioxygenase, *N*-ethylmaleimide reductase, a cytochrome P450, and 2,4-dichlorophenol 6-monooxygenase (Fig. 9; see Data Set S1 in the supplemental material). In particular, homogentisate 1,2-dioxygenase is a ring-cleaving enzyme that has been implicated in the degradation of polycyclic aromatic hydrocarbons, as well as styrene (88). 2,4-Dichlorophenol 6-monooxygenase is a hydroxylase involved in the degradation of chlorinated aromatic pollutants (89, 90). The genes encoding the two subunits of protocatechuate 3,4-dioxygenase, an aromatic-ring-cleaving enzyme implicated in lignin degradation, were also observed in plastic metagenome samples, and the alpha subunit was significantly more abundant in this niche (86). These data only allow for speculation as to whether the microorganisms residing on plastic debris are actually degrading plastic, or cometabolizing adsorbed pollutants. Our data suggest that these plastic-associated microbial communities rely primarily on carbon and other nutrients accumulated by filter-feeding bryozoans, other marine eukaryotes, and autotrophic activity.

In the present study, we applied an integrated approach by focusing on microbial taxonomic and functional composition in the context of the metabolic activity and composition of the entire community. We observed that microplastics create a habitat for metabolically active and net autotrophic communities that may harbor a predictable core group of microbial clades that are functionally distinct from the surrounding picoplankton community in the water column. Future studies aimed at specifically elucidating how natural microbial assemblages interact with plastic might include excluding multicellular eukaryotes. Alternatively, approaches that differentiate microbes growing directly on plastic surfaces from those coassociated with colonizing eukaryotic organisms would help clarify intracommunity biotic interactions occurring on microplastics. Further insights will be gained by comparing microbes on plastic to those on natural surfaces in the open ocean such as driftwood, floating algae, plankton, migratory fish, and other wildlife. Such future work will be useful in further determining how plastic debris may uniquely impact open-ocean community composition, processes, and organism dispersal.

## MATERIALS AND METHODS

The SUPER HI-CAT expedition took place aboard the R/V *Kilo Moana* and transited from Oahu, HI, to California between 25 August and 5 September 2008. Hydrographic and biogeochemical data were collected along the expedition route to characterize the upper 150 m of the water column at discrete depths at each station. Water samples for measurements were collected via 10-liter polyvinyl chloride bottles affixed to a conductivity-temperature-density rosette sampler. In order to quantify neustonic plastic debris, a manta trawl (91) provided by the Algalita Marine Research Foundation with a rectangular opening of 0.9 by 0.15 m, a 3.5-m-long, 333-µm mesh net, and a flowmeter was towed off the stern for ~90 min at a speed of 1 to 2 knots. Upon recovery of the manta trawl, samples were separated into three different size classes with mesh-lined screens: >5 mm, >2 to 5 mm, and 0.2 to 2 mm. With the aid of a dissecting microscope and forceps, we carefully separated identifiable plastic fragments from any natural particles captured by the trawl. Previous studies have used similar approaches and shown that visual inspection with the aid of a dissecting microscope is sufficient to discriminate between plastic and natural particles down to at least 1 mm in diameter (3, 32, 92). In addition, the base polymer of a subset of plastic particles from each size class was identified by FTIR by the analytical chemistry consulting company Analytical Answers (Woburn, MA). The one-dimensional area of the largest surface and length of individual plastic particles (*n* = 554) were determined with ImageJ software (http://rsb.info.nih.gov/ij/), and the total surface area of each plastic particle was then estimated on the basis of the approximate shape of the particle.

### Biotic measurements.

Plastic particle and water column Chl *a* measurements were carried out with a Turner Designs model 10-AU fluorometer and the standard protocol used by the Hawaii Ocean Time-series program (http://hahana.soest.hawaii.edu). Plastic particle Chl *a* values were then normalized to the surface area of individual plastic particles in order to approximate the relationship between Chl concentrations and plastic particle size. Rates of community metabolism were estimated by utilizing light-dark bottle oxygen production and consumption measurements (93, 94). These provided estimates of NCP (the balance of oxygen produced and consumed in the light bottle incubations relative to a time zero value), R (the total oxygen consumption in the dark bottle incubations relative to a time zero value), and GPP (the total production of oxygen, calculated as NCP + R). For water measurements, seawater was collected from near-surface waters (~7 m) and placed into a triple-rinsed 20-liter polycarbonate carboy. Subsamples were incubated as described by Viviani et al. (95). Briefly, 24 125-ml borosilicate iodine bottles were filled with seawater after overflowing 3 full volumes to fully flush out air bubbles. Eight bottles were immediately fixed with Winkler oxygen reagents, eight bottles were placed in an opaque plastic container for incubation in the dark, and eight bottles were incubated in the light. Bottles were incubated for 24 h in surface seawater-cooled incubators, shaded to ~30% surface irradiance.

To assess the community metabolism of organisms associated with plastic particles, 10 to 14 plastic pieces of a given size class (>5 mm, 2 to 5 mm, or 0.2 to 2 mm) were chosen and placed individually into borosilicate iodine bottles filled with seawater. Plastic-amended bottles were then divided and incubated under either light or dark conditions as described above at the same time and with the same seawater as for water column metabolic rate determinations. An effort was made to ensure that the plastic pieces used were similar in terms of color, approximate size (within each size class), and the presence or absence of visible biofilm. Measured oxygen concentrations of bottles containing plastic particles were adjusted to take into account the approximate volume of the water displaced by the plastic pieces. To calculate GPP, NCP, and R for individual plastic particles, background seawater community rates measured from the unamended bottles were subtracted from the rates measured in plastic-amended bottles.

### SEM.

SEM images were taken in 2015 with plastic particles that were fixed in formalin immediately after collection during the SUPER HI-CAT expedition. Formalin-fixed samples were postfixed with 1% OsO_4_ in 0.1 M sodium cacodylate, dehydrated through an ethanol series, and dried in a Tousimis Samdri-795 critical-point dryer. Particles were mounted on aluminum stubs, sputter coated with palladium in a Hummer 6.2 sputter coater, and viewed with a Hitachi S-4800 Field Emission Scanning electron microscope at an accelerating voltage of 5 kV.

### SUPER HI-CAT library construction, sequencing, and annotation.

Immediately after size sorting, individual plastic particles collected for DNA analyses were placed in sterile 2.0-ml microcentrifuge tubes, flash frozen in liquid nitrogen, and stored at −80°C until DNA extraction. To begin extraction, samples were defrosted on ice. Mechanical disruption of cells then took place by a bead-beating approach where 0.1 g of sterile zirconia beads (BioSpec Products, Bartlesville, OK) and 200 µl of 1× Tris-EDTA buffer (pH 8.5) were added to each tube and the tubes were reciprocated (Fast Prep machine; Bio 101, Carlsbad, CA) at setting 6.0 for a total of 2 min (two 45-s run times and one 30-s run time). Afterward, 350 µl of lysis buffer 1 (final concentrations, 50 mM Tris, 20 mM EDTA, 1.2% Triton X-100, 10 g liter^−1^ lysozyme, and 200 mg·liter^−1^ RNase A) was added and samples were shaken at 250 rpm at 37°C for 2 h. Following this, 200 µl of lysis buffer 2 (final concentrations, 1% SDS, 1% potassium xanthogenate, 50 mM Tris, 20 mM EDTA, and 0.65 g liter^−1^ proteinase K) was added and the samples were shaken at 125 rpm for 18 h at 56°C. Xanthogenate disrupts cyanobacterial cell walls and sequesters metal ions (96). Following this, 600 µl of buffer AL from the Qiagen DNeasy Blood and Tissue minikit was added, and samples were processed in accordance with the kit manufacturer’s instructions for DNA purification of bacterial cells. Two wash steps were performed with buffer AWI.

DNA concentrations were quantified with a PicoGreen assay (Invitrogen, Waltham, MA). Metagenomic libraries were constructed with the Illumina TruSeq library preparation protocol including a 2% PhiX spike-in (Illumina, Inc., San Diego, CA). Each library was first sequenced with the Illumina MiSeq system to obtain a preliminary assessment of the plastic-associated communities and then sequenced with the Illumina NextSeq500 system to achieve deeper sequencing depth per library.

For each sequenced library, adaptors were removed with Trimmomatic (97) v. 0.27 (parameters: ILLUMINACLIP::2:40:15) and paired-end reads were then joined with PANDAseq (98) v. 2.4 (parameters: -F 6 −t 0.32). The ends of joined reads and reads unable to be paired with quality scores of <5 were clipped, and sequences shorter than 40 bases or made up of more than 90% of a single base were discarded. Paired-end reads that did not overlap were joined with “NNNNNN” inserted between them so nonoverlapping paired ends would not be double counted in statistical analyses. Low-complexity regions of reads were masked with TANTAN (99). Phage PhiX sequences were identified for removal by mapping reads to the PhiX genome with Bowtie2 (100) v. 2.1.0 (parameters: -local). After trimming and quality filtering, libraries sequenced with the MiSeq and NextSeq systems contained 0.5 to 2.4 million and 14.9 to 44.6 million reads, respectively. SortMeRNA (101) v. 1.7 with the databases Rfam (102) v. 11.0 and SILVA (103) release 111 were then used to separate reads into SSU rRNA gene and non-rRNA gene bins and each bin for MiSeq and NextSeq libraries from the same sample were combined. For the total numbers of reads assigned to SSU rRNA genes and non-rRNA genes, see Table S2 in the supplemental material.

10.1128/mSystems.00024-16.10 Table S2  Metagenomic library information. Download Table S2, DOCX file, 0.1 MB. Copyright © 2016 Bryant et al. 2016 Bryant et al. This content is distributed under the terms of the Creative Commons Attribution 4.0 International license.

Reads identified as containing SSU rRNA genes (here referred to as SSU rRNA gene reads) were then queried against the SILVA SSU Ref database (103) release 119 with Last version 418 (parameters: -n 200 -u 2 -Q 1 -s 2 -m 2500). Bacterial database sequences with a pintail value of <50, indicating a high probability that the sequence is chimeric, and bryozoan database sequences that have been previously identified as chimeric or misannotated were removed from the SILVA database (104). In addition, some sample reads mapped to contaminant non-SSU regions at the ends of several database sequences. Reads mapping to these contaminated regions were removed.

For the remaining reads that mapped to the SILVA database, all hits with a minimum alignment length of 100 bp, a minimum bit score of 50, and a bit score within 1% of the bit score of the best hit (including the best hit) were retained. Each read was then assigned to the lowest common ancestor (LCA) of those retained hits. For example, if an SSU rRNA gene read had two high-scoring hits, each from the same family but a different genus, that read would be assigned to the common family and not given a genus or species assignment. The 1% cutoff was chosen for LCA assignments to allow for high-resolution taxonomic assignments while also considering hits that differed from the query sequence by only a few base pairs less than the top hit. SSU rRNA gene reads mapping to SILVA chloroplast SSU sequences were also annotated by using the PhytoREF database (105) v. 1.1 by the same approach.

To visualize distances between eukaryotic and bacterial communities in each plastic sample, class or family level SSU rRNA gene read counts for eukaryotes or bacteria, respectively, were used to generate NMDS plots by using the metaMDS function and Bray-Curtis distances in the Vegan R package (VEGAN). Reads not able to be assigned to the targeted taxonomic level were removed from this analysis. Subsequently, counts per clade were normalized by calculating their proportions relative to the total number of SSU rRNA gene read counts per sample and rounding proportions to the nearest thousandth (eukaryotes) or hundredth (bacteria) decimal place. Values were then square root transformed. Rounding of the proportions accounted for variability in total read counts between samples, similar to random subsampling of larger libraries down to the sequencing depth of the smallest library (rarefying; *n* = 3,560 [eukaryotes] and *n* = 600 [bacteria]) but without the addition of artificial uncertainty (106).

### Comparison to plastic debris from the North Atlantic Subtropical Gyre.

We downloaded bacterial amplicon sequences from the study conducted by Zettler et al. (29) at the NCBI Sequence Read Archive (SRR907634 to SRR907639). SFF files were processed with the following QIIME v. 1.8.0 scripts (107). We used process_sff.py to convert SFF files into FASTA and QUAL files, split_library.py (parameters: -w 50 -r -l 100 -z truncate_only) to demultiplex reads, denoise_wrapper.py and inflate_denoiser_output.py to denoise the flowgrams, and identify_chimeric_seqs.py (parameters: -m usearch61 −r gg_97_otus_4feb2011.fasta) with filter_fasta.py to identify and remove chimeric sequences. Reads were then annotated with the SILVA database as described above. To control for differing read lengths and taxonomic information across different regions of SSU rRNA genes, we only included reads in the analysis that were able to be assigned a family level clade by the LCA approach.

### Comparison of taxonomic and bacterial functional gene abundances in plastic-associated communities and surrounding picoplankton communities.

Samples used to generate picoplankton (0.22- to 1.6-µm seawater size fraction) 454 metagenomes (NCBI SRA numbers SRX556050 and SRX556052 to SRX556067) were collected at a 25-m depth at Hawaii Ocean Time-series station ALOHA between December 2007 and September 2009 (38). Library preparation and processing have been described previously (38). It has previously been demonstrated that the 454 and Illumina platforms sample the same fraction of diversity and produce similar relative abundances of genes and genomes (63), although 454 does produce fewer reads per sequencing run (addressed below).

SSU rRNA genes in picoplankton libraries were identified and annotated as described above for the SUPER HI-CAT data sets. Since the picoplankton samples were sequenced by the older technology, there were fewer SSU rRNA gene read counts per sample. We used Mann-Whitney U tests and the Benjamini-Hochberg procedure for FDR correction (FDR, <0.005) to test for differential abundances of microbial families between the two communities, as recommended for comparing categories of samples with uneven library sizes (108). To account for differing read depths between samples, we calculated the proportion of SSU rRNA gene read counts to each prokaryotic family in each sample, relative to the total SSU rRNA gene read counts to all prokaryotic families per sample and rounded these proportions to the hundredth decimal place (approximating rarefying to ~200 reads).

In order to target bacterial protein-coding genes, low-complexity regions of reads from the SUPER HI-CAT and picoplankton data sets were masked with TANTAN (99) and then compared to NCBI RefSeq database 69 with Last (parameters: -b 1 -x 15 -y 7 -z 25 -F 15 -u 2 -m 10 -Q 0), including the default 1e-06 E value cutoff. Reads were considered to originate from bacterial cells if all of the best-scoring hits, with an alignment length of at least 50 amino acids, were to bacterial genomes. These reads were queried against the KEGG database (109; accessed 4 April 2014) with Last (parameters same as above). Reads were assigned to the KO annotation of their top LAST hit. This produced the same results as adding an additional bit score 50 requirement.

To test for KO with a >2 log_2_-fold difference in abundance (log_2_ change, >2-fold; FDR, <0.005) between the picoplankton and SUPER HI-CAT bacterial communities, we used DESeq2 (110). In brief, the DESeq2 algorithm uses negative binomial generalized linear models to test for differential abundances in count data and estimates size factors to control for variation in sequencing depth between libraries. It applies the Benjamini-Hochberg procedure to account for multiple comparisons. To confirm that KOs identified as differentially abundant by DESeq2 are not false positives due to uneven library sizes between the picoplankton and SUPER HI-CAT data sets, we also transformed KO read counts to proportions relative to the total number of reads assigned to a KO, rounded proportions to the fifth decimal place (approximating rarefying to ~216,000 reads), and applied Mann-Whitney U tests, followed by the Benjamini-Hochberg procedure (FDR, <0.005).

### Nucleotide sequence accession numbers.

Illumina TruSeq and NextSeq500 metagenomic libraries generated for this study were deposited in the NCBI SRA (https://www.ncbi.nlm.nih.gov/sra) under BioProject number PRJNA318384 with sample accession numbers SRS1401924 to SRS1401935 (see Table S2 in the supplemental material).
