# Supplementary material for: Diversity and Activity of Communities Inhabiting Plastic Debris in the North Pacific Gyre
Source: mSystems. 2016 May 17;1(3):e00024-16. doi: 10.1128/mSystems.00024-16 (PMC5069773; doi:10.1128/mSystems.00024-16)
Supplement: Table S1 [file sys003162023st1.docx]

**Table S1.** Sampling station information and summary of plastic concentrations in the >2-5 mm and > 5 mm size classes at each station in the North Pacific Subtropical Gyre.

| **Station** | **Latitude** | **Longitude** | **Collection Date** | | | **2-5 mm, # m^-3^** | | | **>5 mm, # m^-3^** | | | **Total Areal Abundance, # km^-1^** | | |
| --- | --- | --- | --- | --- | --- | --- | --- | --- | --- | --- | --- | --- | --- | --- |
| STN-2 | 27° 43.795 N | 154° 53.897 W | 27-Aug-08 | | 0.22 | | | 0.12 | | | 51,000 | |  |  |
| STN-3 | 28° 35.042 N | 154° 34.317 W | 27-Aug-08 | | 0.28 | | | 0.07 | | | 52,500 | |  |  |
| STN-4 | 31° 12.787 N | 153° 29.632 W | 28-Aug-08 | | 1.53 | | | 0.25 | | | 267,000 | |  |  |
| STN-5 | 31° 59.450 N | 153° 03.452 W | 28-Aug-08 | | 0.85 | | | 0.11 | | | 144,000 | |  |  |
| STN-6 | 34° 05.050 N | 151° 37.916 W | 29-Aug-08 | | 0.78 | | | 0.11 | | | 133,500 | |  |  |
| STN-7 | 34° 28.125 N | 151° 19.283 W | 29-Aug-08 | | 0.68 | | | 0.11 | | | 118,500 | |  |  |
| STN-8 | 34° 38.423 N | 150° 45.928 W | 29-Aug-08 | | 2.3 | | | 0.19 | | | 373,500 | |  |  |
| STN-9 | 34° 55.675 N | 148° 01.812 W | 30-Aug-08 | | 1.2 | | | 0.31 | | | 226,500 | |  |  |
| STN-10 | 34° 59.026 N | 147° 24.349 W | 30-Aug-08 | | 3.03 | | | 0.68 | | | 556,500 | |  |  |
| STN-11 | 35° 02.066 N | 146° 48.626 W | 30-Aug-08 | | 1.19 | | | 0.24 | | | 214,500 | |  |  |
| STN-12 | 35° 14.797 N | 143° 47.303 W | 31-Aug-08 | | 1.4 | | | 0.25 | | | 247,500 | |  |  |
| STN-13 | 35° 18.046 N | 142° 47.506 W | 31-Aug-08 | | 1.63 | | | 0.13 | | | 264,000 | |  |  |
| STN-14 | 35° 25.376 N | 139° 15.016 W | 1-Sep-08 | | 1.41 | | | 0.19 | | | 240,000 | |  |  |
| STN-15 | 35° 26.331 N | 138° 15.861 W | 1-Sep-08 | | 1.02 | | | 0.12 | | | 171,000 | |  |  |
|  |  |  |  |  | | |  | | |  | | | |  |
|  | | | | | | | | | | | | | |  |
