# Supplementary figures and images for: Diversity and Activity of Communities Inhabiting Plastic Debris in the North Pacific Gyre
Source: mSystems. 2016 May 17;1(3):e00024-16. doi: 10.1128/mSystems.00024-16 (PMC5069773; doi:10.1128/mSystems.00024-16)

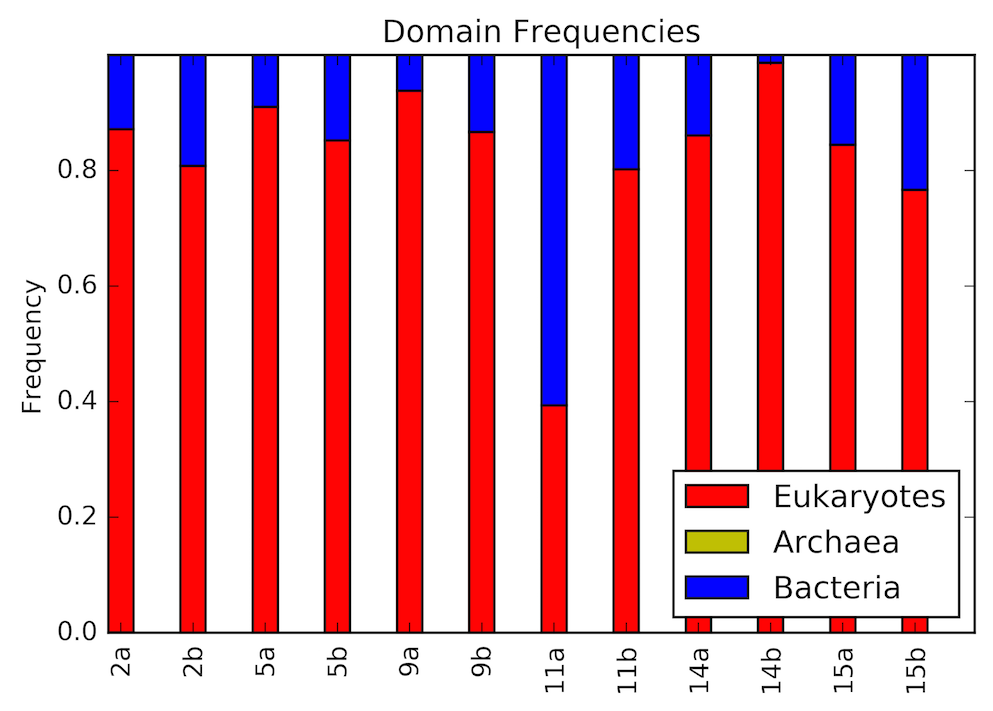

Supplement: Figure S1 [file sys003162023sf3.tif]

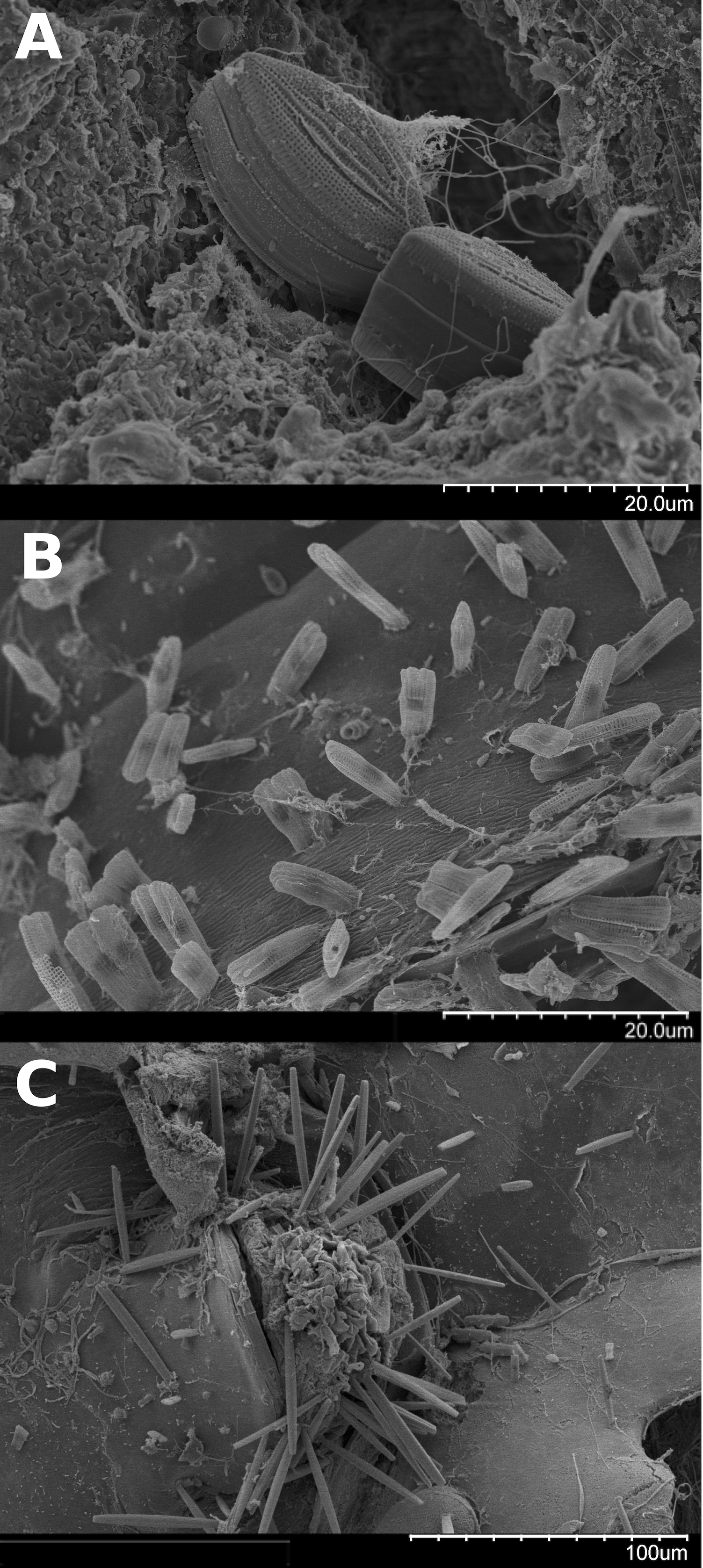

Supplement: Figure S3 [file sys003162023sf5.tif]

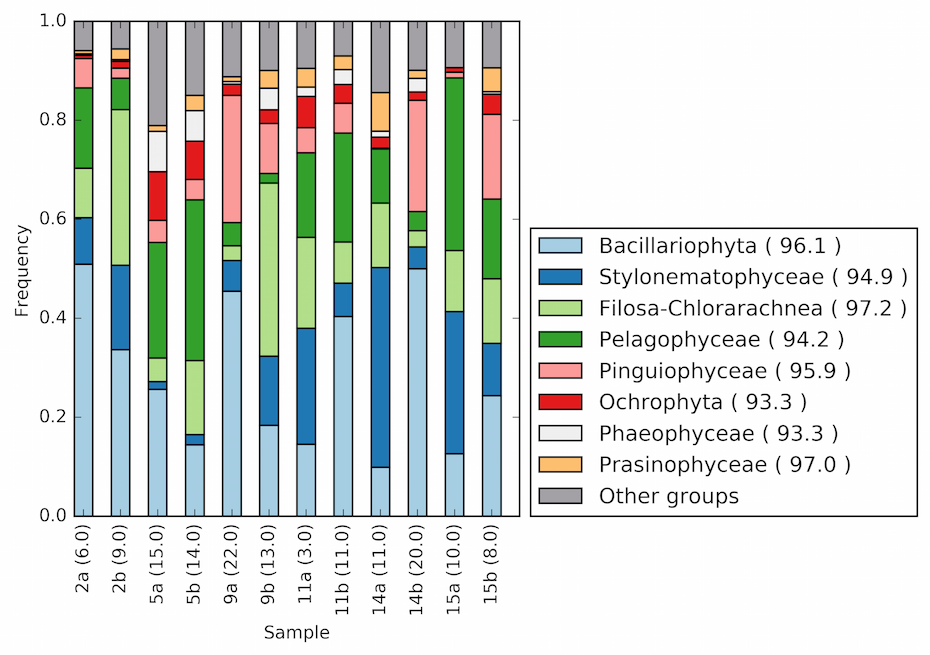

Supplement: Figure S4 [file sys003162023sf6.tif]

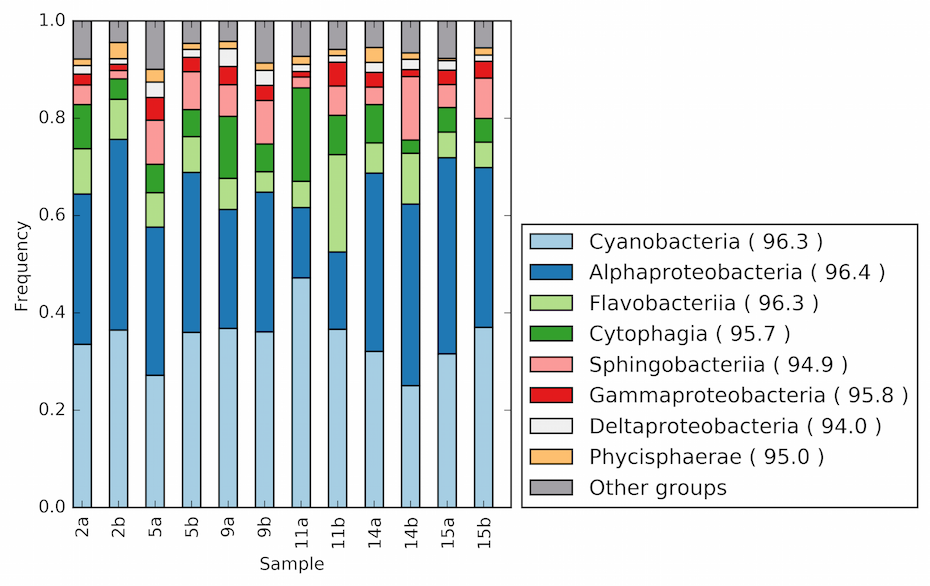

Supplement: Figure S5 [file sys003162023sf7.tif]

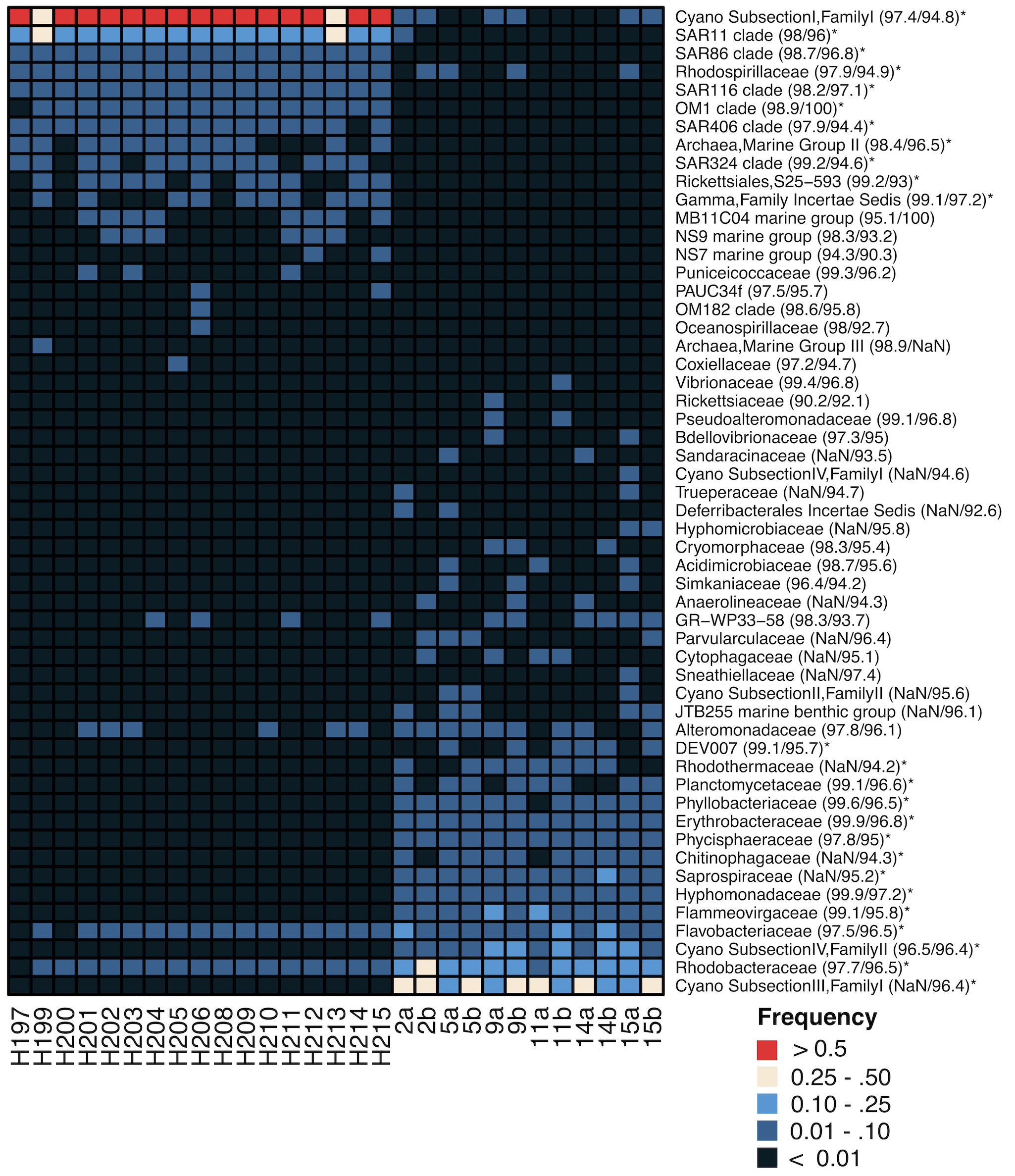

Supplement: Figure S6 [file sys003162023sf8.tif]

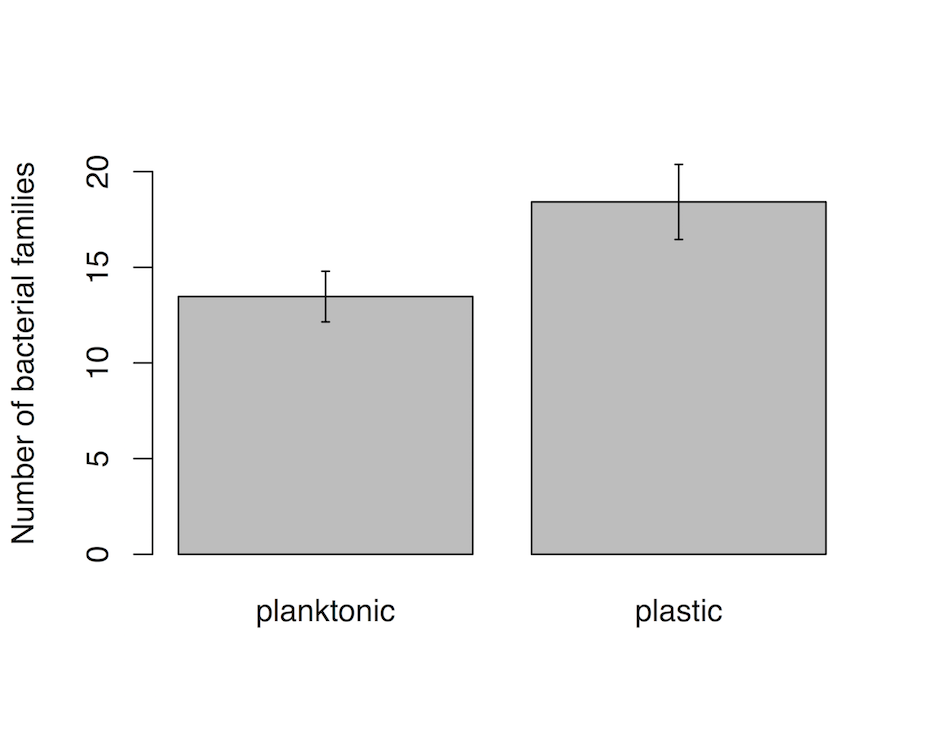

Supplement: Figure S7 [file sys003162023sf9.tif]
