# Supplementary material for: Diversity and Activity of Communities Inhabiting Plastic Debris in the North Pacific Gyre
Source: mSystems. 2016 May 17;1(3):e00024-16. doi: 10.1128/mSystems.00024-16 (PMC5069773; doi:10.1128/mSystems.00024-16)
Supplement: Table S2 [file sys003162023st2.docx]

**Table S2.** Metagenomic library information.

| **Sample Name** | **Station #** | **Size Class (mm)** | **NCBI Sample Accession Numbers** | **Number of SSU rRNA fragments*** | **Number of non-rRNA reads*** | **Number of reads mapping to bacterial coding sequences in RefSeq*** | |  |
| --- | --- | --- | --- | --- | --- | --- | --- | --- |
| 2a | STN-2 | >5 | SRS1401934 | 38,691 | 22,897,674 | 2,521,340 | |  |
| 2b | STN-2 | >2-5 | SRS1401935 | 33,112 | 27,103,800 | 3,843,816 | |  |
| 5a | STN-5 | >5 | SRS1401933 | 62,001 | 46,784,460 | 2,779,836 | |  |
| 5b | STN-5 | >2-5 | SRS1401932 | 7,941 | 31,300,709 | 687,320 | |  |
| 9a | STN-9 | >5 | SRS1401931 | 62,941 | 45,214,833 | 1,821,896 | |  |
| 9b | STN-9 | >2-5 | SRS1401930 | 30,629 | 38,649,677 | 2,216,048 | |  |
| 11a | STN-11 | >5 | SRS1401929 | 9,006 | 15,331,390 | 2,613,731 | |  |
| 11b | STN-11 | >2-5 | SRS1401927 | 15,660 | 20,055,718 | 1,559,515 | |  |
| 14a | STN-14 | >5 | SRS1401926 | 40,253 | 46,178,956 | 3,420,774 | |  |
| 14b | STN-14 | >2-5 | SRS1401928 | 50,737 | 28,251,664 | 381,636 | |  |
| 15a | STN-15 | >5 | SRS1401925 | 19,245 | 38,613,467 | 1,860,711 | |  |
| 15b | STN-15 | >2-5 | SRS1401924 | 20,959 | 20,485,893 | 2,638,598 | |  |
| *Read statistics after quality trimming and paired-end assembly of combined MiSeq and NextSeq500 DNA sequencing data. | | | | | | |  | |
